# Pneumatosis cystoides intestinalis linked to sunitinib treatment for renal cell carcinoma

**DOI:** 10.1002/iju5.12119

**Published:** 2019-09-17

**Authors:** Hiroyuki Shikuma, Shogo Inoue, Tomoya Hatayama, Sakurako Mukai, Masayuki Muto, Shunsuke Miyamoto, Kosuke Sadahide, Shinsuke Fujii, Yohei Sekino, Keisuke Goto, Shunsuke Shinmei, Keisuke Hieda, Tetsutaro Hayashi, Jun Teishima, Akio Matsubara

**Affiliations:** ^1^ Department of Urology Hiroshima University Hospital Hiroshima Japan

**Keywords:** operation, pneumatosis cystoides intestinalis, sunitinib

## Abstract

**Introduction:**

Pneumatosis cystoides intestinalis is a rare condition characterized by air‐filled cysts within intestinal walls. It can be caused by various factors. We report a case of pneumatosis cystoides intestinalis linked to sunitinib treatment for renal cell carcinoma.

**Case presentation:**

A 67‐year‐old female with advanced renal cell carcinoma who had been treated with sunitinib visited our hospital complaining of abdominal pain. Computed tomography scans showed diffuse air‐filled cystic formation of intestine. We treated with conservative therapy, and she recovered. However, although air‐filled cysts disappeared in the images, intraoperative findings in the resection of a recurrent paracaval lymph node showed a thinning of the intestine.

**Conclusion:**

It is necessary to consider pneumatosis cystoides intestinalis when a patient using a tyrosine kinase inhibitor complains of abdominal symptoms. It should also be noted that the effect of pneumatosis cystoides intestinalis may remain even if pneumatosis disappears from the image on tomography scans.

**Abbreviations & Acronyms**
CT: computed tomography
PCI: pneumatosis cystoides intestinalis
RCC: renal cell carcinoma
TKI: tyrosine kinase inhibitor
VEGF: vascular endothelial growth factor

**Keynote message**

We report a case of PCI related to sunitinib in which the patient recovered after conservative therapy. We found that the influence of PCI might persist even if the pneumatosis disappears from the images on tomography scans. For this reason, clinicians should take thorough follow‐up care with patients exhibiting PCI linked to TKI.

## Introduction

PCI is characterized by air‐filled cysts within intestinal walls. It can be caused by infection, intestinal obstruction, intestinal ischemia, and so on.^1^ Sunitinib is a multi‐TKI that is known to have some adverse effects.^2^, ^3^ PCI is a rare adverse effect of sunitinib^4^ and is characterized by the presence of gas in the submucosa or subserosa of the intestinal wall.^5^ Here, we report a case of PCI in a patient who was treated with TKI. PCI disappeared on the image due to conservative treatment, but at the time of a later surgery, a thinned intestinal wall caused by PCI was recognized.

## Case presentation

A 67‐year‐old female visited our hospital due to a right renal tumor. Her past medical history is hypertension, dyslipidemia, diabetes, and rectal cancer. Her medication was amlodipine, telmisartan, atorvastatin, glimepiride, and sitagliptin. The CT scan showed a right renal tumor and an enlarged paracaval lymph node. We diagnosed RCC (T1aN1M0) and performed open nephrectomy and lymph node dissection. The pathological diagnosis was clear cell RCC with a sarcomatoid carcinoma component.

**Figure 1:**
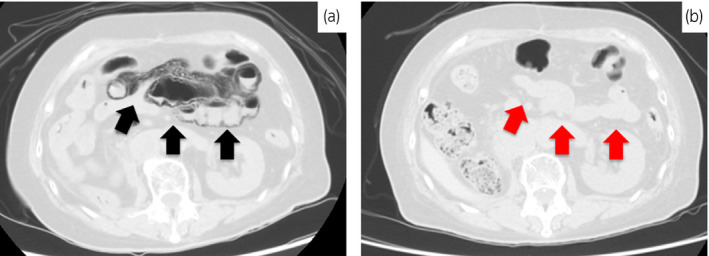
CT performed at the time of PCI diagnosis and on follow‐up. (a) CT performed to detect cause of vomiting. PCI is seen in a wide area around the ileocecal region (black arrow). There is no free gas or ascites. (b) CT on 7th day after discontinuation of sunitinib. PCI is reduced (red arrow).

A subsequent CT scan 66 days after the operation showed a paracaval lymph node recurrence. We administered sunitinib immediately and then started treatment at a dose of 50 mg/day (4 weeks out of 6) and then 25 mg/day (4 weeks out of 6) for hand‐foot syndrome. After 301 days of treatment, the patient visited our hospital reporting nausea, vomiting, and abdominal pain. Her general condition was good, and her vital signs were normal. There was no peritoneal irritation in the physical findings. Laboratory findings indicated no problems. A CT scan showed a diffuse air‐filled cystic formation in the intestine but no pneumoperitoneum (Fig. 1a). We diagnosed PCI linked to sunitinib and admitted her to receive conservative management; we administered an intravenous drip extracellular fluid, and also stopped the sunitinib treatment. After hospitalization, her symptoms showed signs of improvement and 7 days later, a follow‐up CT showed lost of intestinal wall gas, so she was discharged (Fig. 1b).

**Figure 2:**
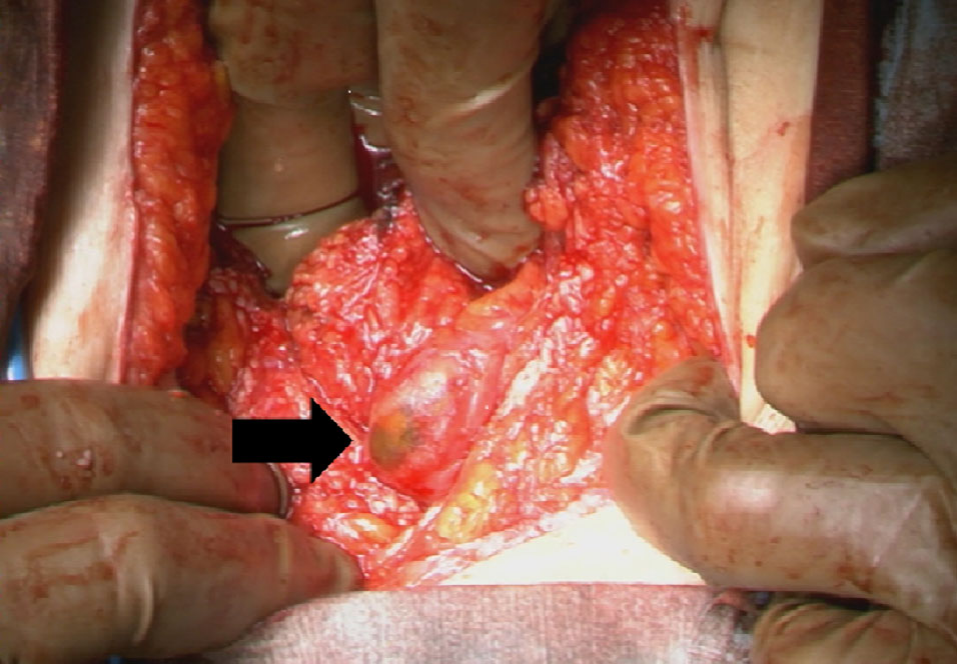
Intraoperative finding of PCI. A portion of the intestine contents can be seen through the thin serosa (black arrow).

Forty‐two days after PCI treatment, we planned a surgical resection of the recurrent paracaval lymph node. After successful resection of the node, we examined the intestinal tracts and there was no perforation. However, a portion of the intestinal tract exhibited a thin serosa through which we could see the contents of the intestinal tract (Fig. 2). We assumed that it was caused by PCI, and we sutured it for reinforcement. The postoperative course was good and the patient was discharged postoperative day 7. After surgery, paracaval lymph nodes appeared again and treatment with nivolumab was started.

## Discussion

PCI is caused by air‐containing cysts in the wall of the intestinal tract. Although the pathogenesis has not been fully established, various causes or clinical situations have been suggested to explain the development of PCI.^6^

The precise mechanism of PCI linked to TKI is still unknown. Sunitinib is a multi‐TKI and has an antitumor effect by means of VEGF‐receptor inhibition. However, sunitinib acts on the capillaries of small intestinal villus, and as such, decreases the blood vessel density of intestinal mucosa and reduces mucosal regeneration, which leads to microperforation and the occurrence of PCI.^7^, ^8^, ^9^

PCI linked to the treatment of sunitinib for RCC has also been reported (Table 1). In the case presented in this work, there were no co‐morbidities or medications that could cause PCI, and there were no infections or mechanical triggers, so we judged it to be sunitinib. In many cases, PCI with sunitinib occurs within 1 year of administration, and this case occurred 10 months after the start of administration, but it has also been known to occur after more than 2 years, so care must be taken with long‐term administration.

**Table 1:** Previously reported PCI due to sunitinib

Author	Age/sex	Symptoms	Location	Perforation	Duration of treatment (m)	Treatment	Outcome	Resume sunitinib
Coriat *et al*.^12^	68/M	None	Small intestine	−	28	Operation	Resolved	Yes (after 7 months)
Choi *et al*.^13^	66/M	Abdominal pain	Small intestine	+	3	Operation	Resolved	NA
Flaig *et al*.^4^	NA/F	Flank pain	Colon	+	13	Operation	NA	NA
Flaig *et al*.^4^	NA/F	Abdominal pain, diarrhea	Colon	−	14	Observation	Resolved	NA
Shinagare *et al*.^14^	68/M	None	Small intestine	−	3	Observation	Resolved	NA
Shinagare *et al*.^14^	NA	Abdominal pain	Stomach, small intestine, colon	+	1	Observation	NA	NA
Lee *et al*.^11^	68/F	Diarrhea	Small intestine	−	4	Observation	Resolved	No
Our case	67/F	Abdominal pain, vomiting	Small intestine	−	11	Observation	Resolved	No

PCI treatments include both surgical therapies and conservative therapies. Conservative management includes simple observation, hyperbaric oxygen therapy, parenteral nutrition, and administration of antibiotics. Surgery is needed when PCI is complicated due to perforation, for the management of primary conditions such as intestinal ischemia or obstruction, or when conservative therapy fails. The exclusion of conditions that require surgical treatment is important to avoid unnecessary surgery.^10^ In cases featuring perforation, there is a tendency for surgical therapy to be selected, but in some cases, only conservative treatment will improve conditions even if perforation is present. Surgical exploration is occasionally performed because PCI mimics bowel necrosis or panperitonitis. Since such an operation is performed in the emergency setting, an adequate washout period for TKI is not possible, and that leads to postoperative complications such as delayed wound healing or hemorrhage.^11^ In cases with only minor perforation and no evidence of peritonitis, it is better to use conservative treatment at first. Because there were no symptoms of perforation, our patient was closely observed with conservative management and the indications of PCI immediately disappeared in CT. However, even if PCI disappears from an image, we understand from the intraoperative findings of this case that PCI might not have been completely improved due to the persistent influence of TKI. Moreover, even if it is improved once with conservative treatment, it seems necessary to follow the patient intensively. Prompt identification of sunitinib as the cause is important and clinicians must be aware of this rare adverse effect. Although there have also been cases in the literature in which sunitinib was resumed after PCI, we decided not to restart sunitinib in the present patient, considering the risk of recurrent PCI.

Although PCI is a very rare side effect of sunitinib, early diagnosis of PCI linked to sunitinib is crucial for avoiding unnecessary surgical intervention. Clinicians should be aware of this rare adverse effect and take intensive care for patients with PCI linked to TKI.

## Conclusion

PCI is a rare adverse effect of sunitinib treatment. The influence of PCI may persist, even if the evidence of it has disappeared from a tomography scan, so it is necessary to ensure careful follow‐up.

## Conflict of interest

The authors declare no conflict of interest.
